# Ultrasound for Early Detection of Joint Disease in Patients with Hemophilic Arthropathy

**DOI:** 10.3390/jcm6080077

**Published:** 2017-07-31

**Authors:** Matteo Nicola Dario Di Minno, Gianluigi Pasta, Sonia Airaldi, Federico Zaottini, Antonio Storino, Ernesto Cimino, Carlo Martinoli

**Affiliations:** 1 Department of Advanced Biomedical Sciences, Federico II University, 80131 Naples, Italy; 2 Dipartimento di Ortopedia—Fondazione IRCCS Ca’ Granda, Ospedale Maggiore Policlinico, 20122 Milan, Italy; gianluigipasta@yahoo.it; 3 Radiologia III—IRCCS San Martino-IST—DISSAL, Università di Genova, 16132 Genova, Italy; airaldi.sonia@gmail.com (S.A.); federico.zaottini.fz@gmail.com (F.Z.); mskeletal.radiology@gmail.com (C.M.); 4 Department of Public Health, Federico II University, 80131 Naples, Italy; antonio_storino@virgilio.it; 5 Department of Clinical Medicine and Surgery, Federico II University, 80131 Naples, Italy; ernesto.cimino@unina.it

**Keywords:** hemophilia, hemophilic arthropathy, ultrasound, imaging

## Abstract

Joint bleeding represents the most commonly reported type of hemorrhage in patients affected by hemophilia. Although the widespread use of prophylaxis has been able to significantly reduce the onset of arthropathy, it has been shown that a non-negligible percentage of patients develop degenerative changes in their joints despite this type of treatment. Thus, periodic monitoring of the joint status in hemophilia patients has been recommended to identify early arthropathic changes and prevent the development or progression of hemophilic arthropathy. Ultrasound (US) has proven able to detect and quantify the most relevant biomarkers of disease activity (i.e., joint effusion and synovial hypertrophy) and degenerative damages (i.e., osteo-chondral changes) by means of scoring scales of increasing disease severity. In the present review, we have detailed major literature evidence about the use of US to assess joint status in hemophilia patients, focusing on signs of disease activity and degenerative damages. In particular, we have discussed recent evidence about “point-of-care” use patients with hemophilia.

## 1. Introduction

Joint bleeding represents the most commonly reported type of hemorrhage in patients affected by hemophilia [1,2,3]. Repeated bleeding episodes may lead to degenerative arthropathy, that is the most frequent complication in patients with both severe and moderate hemophilia [4,5,6,7,8]. Although the widespread use of prophylaxis has been able to significantly reduce the onset of arthropathy, it has been shown that a non-negligible percentage of patients develop degenerative changes in their joints despite this type of treatment [4,5]. With the aim to identify early arthropathic changes and prevent the development or progression of hemophilic arthropathy, periodic monitoring of the joint status in hemophilia patients has been recommended in the framework of comprehensive care [9,10]. Considering that the sensitivity and specificity of physical examination assessment scores (e.g., Gilbert Orthopedic Joint Score—WFH—and the Hemophilia Joint Health Score—HJHS) remain challenging in the identification of early, subclinical joint abnormalities, and that the severity of joint impairment could be missed [5,11,12,13], the use of radiography and magnetic resonance (MR) imaging has been recommended as a complement to clinical examination for assessing the joint status and following the disease progression in hemophilia patients [14]. However, radiography is able to detect advanced arthropathic changes, but has a poor value in recognizing early disease signs [15,16]. On the other hand, MR imaging can be considered highly sensitive to reveal signs of disease activity and effective to perform a comprehensive evaluation of the joint surfaces, but it cannot evaluate more than one joint in a single study, the examination time cannot be shorter than 25–30 min per joint to get accurate information on the status of the articular surfaces, joint positioning in the magnet may be difficult in advanced osteoarthritis, especially at the elbow level, and uncomfortable for the patient [17]. In addition, MR imaging may require sedation in children, is a high-cost modality with long-waiting lists, cannot be used for serial follow-up studies and, in absence of joint effusion, needs intraarticular contrast injection to depict initial osteochondral changes with accuracy [17]. Although often regarded as the imaging modality of choice for the musculoskeletal system, this technique is not suited to the characteristics of hemophilic arthropathy and cannot be used as a screening method for multijoint assessment and repeated follow-up examinations [18,19]. 

## 2. Ultrasound and Disease Activity 

**Figure 1:**
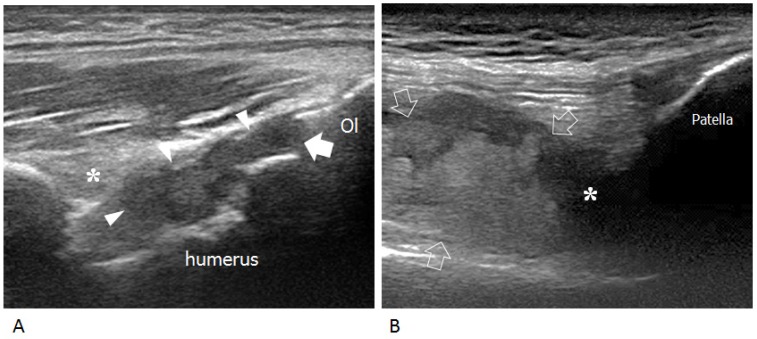
Intraarticular chronic synovial proliferation. (**A**) Longitudinal US image of the posterior elbow demonstrates synovial hypertrophy distending the olecranon recess (arrowheads) and elevating the posterior fat pad (asterisk). The joint line (arrow) is delimited by the olecranon (Ol) and posterior aspect of the humeral trochlea. (**B**) Longitudinal US image of the anterior knee shows marked distension of the suprapatellar recess by synovial hypertrophy (arrows) and effusion (asterisk).

Ultrasound (US) has been proven capable of detecting and quantifying the most relevant biomarkers of disease activity (i.e., joint effusion and synovial hypertrophy) and degenerative damages (i.e., osteo-chondral changes) by means of scoring scales of increasing disease severity. In recent years, six scoring systems based on US have been proposed to quantify joint abnormalities in patients with hemophilia [10,20,21,22,23,24,25], all of which were designed with the final goal of implementing US as part of the diagnostic workup and for monitoring hemophilic arthropathy (Table 1). US would have the intrinsic value of improving identification of subclinical conditions as well as improving workflow due to its availability and portability, thus limiting the number of MR imaging examinations to specific indications. Since 1987, when Wilson et al. [26] described the potential value of US to assess acute hemarthrosis in a series of 38 hemophilic patients, several studies have been conducted to define the ultimate role of joint US to diagnose acute bleeding episodes within joints and muscles, and establish how to detect blood in the joint cavity, measure its amount, and follow up its reabsorption until complete disappearance [20,27,28,29]. US has proven helpful in distinguishing between inflammatory (serous) effusion from hemarthrosis and in defining whether acute pain episodes in hemophilia patients are related to a bleed or to arthritis-mediated conditions [27,30]. In the evaluation of 40 joints of 30 patients presenting acute pain episodes, Ceponis et al. [27] showed that US was able to redirect the diagnostic thinking in >70% of episodes, suggesting that significant discrepancies exist between US findings and patient/physician perceived pain classification as bleeding. Similarly, Aznar et al. [31] reported 37 cases of suspected hemarthrosis evaluated with a home-delivered US assessment. In 16% of cases, US did not show intraarticular blood, thus suggesting arthritis-related pain. In these cases, replacement treatment was discontinued with significant cost savings. On the other hand, in patients with acute hemarthrosis, the replacement therapy was continued until US depicted disappearance of the effusion [31]. As a whole, these data suggest that the use of US could improve both diagnosis and management of acute bleeding episodes in hemophilia patients [20,30]. Although joint effusion can be regarded as an indicator of acute hemarthrosis [30], this sign is a transitory fluctuating parameter and cannot express the status of a joint [32]. Outside the context of an acute bleeding episode, US has proven to be an excellent diagnostic tool to assess synovial hypertrophy and osteochondral changes in joints that are almost totally asymptomatic (Figure 1) [10,33]. The occurrence of synovial hypertrophy plays a pivotal role in the pathogenesis of blood-induced joint damage, activating an auto-catalytic system [34,35,36,37,38]. Given that, synovial hypertrophy is a parameter that can be taken into account for definition of disease activity in hemophilic joints. Several studies consistently showed that US has very high sensitivity for detection of synovial hypertrophy, with results comparable to MR imaging [33,39,40]. Some scoring scales also include assessment of synovial hyperemia [22,23,24,25], defined as a intrasynovial detection of blood flow signals at color [23,25] or power-Doppler (PD) imaging [22,24]. In rheumatoid arthritis and other chronic inflammatory joint diseases, Doppler techniques proved to be valuable to detect hypervascular patterns as a hallmark of acute inflammation and active disease [41,42]. Borrowing the example of rheumatoid arthritis, some authors proposed Doppler imaging as a means to diagnose and monitor disease activity in hemophilic arthropathy [18,43]. However, intrasynovial Doppler positivity is uncommonly observed in hemophilic patients and, in the rare positive cases, only a few “flags” are visualized, suggesting mild hypervascularity that cannot be considered a pivotal sign to redirect patient’s management [33]. In addition, a high variability in the interpretation of Doppler images, the need for high-end machines to get better performance, and a high interequipment variability is expected with the use of Doppler techniques. Contradictory results are reported in literature on the ability of US to detect intraarticular deposition of hemosiderin. Some authors described some distinctive echotextural features between hemosiderin and synovium, but their statements do not appear substantiated and are contradicted by the fact the hemosiderin is embedded within synovium [21,24,39]. On the other hand, other authors did not find any difference between the US appearance of hemosiderin-laden and hemosiderin-free synovium [44,45]. Similarly, they did not observe any findings that differentiate proliferating synovial tissue in hemophilic joints from the one observed in other chronic joint disorders [46]. In terms of clinical relevance, detection of synovial hypertrophy, regardless its degree of detectable vasculature, represents a sign of undertreatment, possibly related to an insufficient treatment regimen or a limited patient’s compliance.

## 3. Ultrasound and Osteochondral Damage

**Figure 2:**
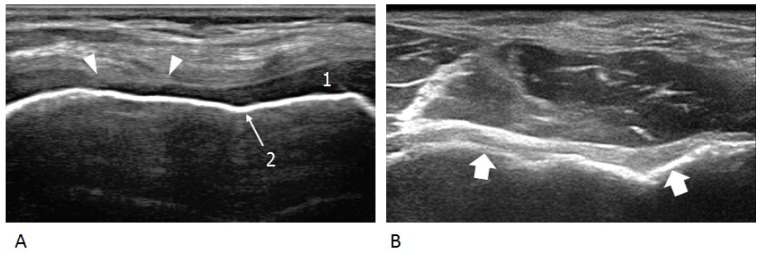
Osteochondral abnormalities. (**A**) Early damage. Transverse US image over the anterior ankle reveals focal partial-thickness loss (arrowheads) of the cartilage (1) investing the talar dome. The subchondral bone (2) retains a normal appearance. (**B**) Advanced damage. Transverse US image over the anterior aspect of the distal humeral epiphysis demonstrates complete loss of the articular cartilage and mild irregularities of the subchondral bone (arrows).

As a result of bleeding episodes, intraarticular hemosiderin deposition induces a pro-inflammatory state, leading to articular cartilage damage and chronic proliferation of synovial tissue that in turn releases lytic enzymes, leading to additional damage to the cartilage and subchondral bone [47,48,49,50]. When osteochondral surfaces are exposed to the US beam, US is very sensitive to detect abnormalities of the articular cartilage and subchondral bone, even if arthropathy is initial and still localized (Figure 2) [51]. Signs of joint derangement represent a major clinical item when assessing patients with hemophilia [39]. In regard to the articular cartilage, US is able to detect the full spectrum of abnormalities, from subtle echotextural changes or partial thickness losses through extensive cartilage disappearance [10]. In children, coexisting damage of the epiphyseal cartilage can be recognized. Concerning subchondral bone, focal and diffuse surface irregularities (incl. cobblestone patterns), erosions, osteophytes, and other overgrowths of bone can be regarded as pathognomonic signs of severe arthropathy [24,52]. A more in-depth evaluation of joints, however, important drawbacks related to problems of access of the US beam. Large part of the weight-bearing areas, the osteochondral surfaces located centrally in the joint cavity and the medullary bone (incl. subchondral cysts) cannot be visualized and this makes US much less comprehensive than MR imaging in providing detailed information about the joint status [21,33,39]. Owing to the diffuse osteochondral involvement, however, such a limited evaluation does not seem to impact significantly on the sensitivity of the method to detect occurrence and assess severity of hemophilic arthropathy [53]. Compared to radiography, US has demonstrated higher sensitivity to detect early damage signs [24]. In addition, good correlation was observed between US and MR imaging in the evaluation of bone erosions and cartilage abnormalities in the elbows, knees, and ankles [33,40]. 

## 4. “Point-of-Care” US and the HEAD-US Architecture

Although a total of six scoring systems [10,20,21,22,23,24,25] have been proposed to implement US in the frame of the diagnostic workup for monitoring hemophilic arthropathy (Table 1), most of them [20,21,22,23,24,25] have been designed to be used by expert sonologists (radiologists or rheumatologists). This type of US approach has been found to be time consuming, requiring about 20 min for each joint assessment [54] and this would make US unlikely to be used in daily practice by hemophilia treaters for screening purposes and in guiding the decision making process. Over recent decades, the development and refinement of simple-to-use, low-cost, portable US machines, with adequate technology to examine both superficial and deep body areas with high-resolution, has promoted the expansion of “point-of-care” use of US in a variety of clinical settings [55]. Point-of-care use means US performed and interpreted by clinicians with the aim to provide a focused decision-making strategy to answer specific clinical questions and identify relevant biomarkers, without the need for a detailed and comprehensive radiological assessment. Point-of-care US is, therefore, not comparable with an US examination performed by imaging specialists (e.g., musculoskeletal radiologists or rheumatologists), but rather supports a more time-efficient, straightforward approach to relevant clinical issues that may affect patient management and treatment strategy. Hemophilic arthropathy is a disease type with great potential of implementation of point-of-care US for routine joint screening. The HEAD-US (Hemophilia Early Arthropathy Detection with UltraSound) system has been designed as a fast-to-perform technique (examination time < 2 min per joint), capable of screening six joints (the elbows, knees, and ankles) in a single examination and recognize biomarkers reflecting disease activity and osteochondral damage [10]. The method includes systematic evaluation of the main recesses of the elbow (i.e., radial, coronoid, annular, olecranon), knee (i.e., suprapatellar, parapatellar), and ankle (i.e., anterior and posterior recesses of the tibiotalar and subtalar joints) to get high sensitivity in detection of joint effusion and synovial proliferation (disease activity items). For osteochondral damage, the HEAD-US method evaluates one osteochondral surface per joint (i.e., anterior aspect of the distal humeral epiphysis in the elbow, femoral trochlea in the knee and anterior aspect of the talar dome in the ankle), assuming that the diffuse derangement of the articular cartilage and subchondral bone that occurs in hemophilic arthropathy may warrant the policy of considering one osteochondral surface only as representative of the overall status of the joint without significantly reducing the sensitivity of the method [10]. The HEAD-US technical guidelines work well to assess the joint status outside the context of an acute bleeding episode, to detect occult or manifest acute bleeding episodes, as well as to assess joints in both adults and children. Joint abnormalities can be quantified using an additive scoring scale that includes items related to disease activity (i.e., hypertrophic synovium) and damage (i.e., cartilage and subchondral bone). Scoring is based on pattern recognition analysis avoiding measurements and the interpretation of findings has been standardized to reduce interoperator variability [56]. The joint assessment based on the HEAD-US system can be accomplished with portable US machines without any need for high-end or proprietary technology and can be learned by non-imaging specialists after a short period of training [57]. It has been proposed that, in daily practice, the HEAD-US system would find its place as a supplement to physical examination assessment tools, such as the Hemophilia Joint Health Score (HJHS), in order to provide more objective assessment of findings and increased sensitivity in detecting joint abnormalities [57]. In this setting, a strong correlation (*r* = 0.88) was observed between HJHS and HEAD-US in the evaluation of the three joints of interest [58]. Interestingly, HEAD-US was able to reveal a higher percentage of abnormalities than HJHS in the children age group [59,60]. In addition, HEAD-US identified synovial hypertrophy even in joints without signs of swelling on HJHS, thus suggesting that US might be more sensitive than HJHS to detect signs of disease activity and subclinical bleeds [58]. Detection of initial asymptomatic damage was also found in an unexpectedly high percentage of cases. 

As a further aspect to consider, although intra-observer variability using US may be a potential issue, all available studies [24,33,39] consistently show a 97% repeatability of US assessment.

## 5. Perspectives

In our expectations, the use of US as part of routine clinical examination by hemophilia specialists would optimize the diagnostic workflow avoiding additional costs and long waiting lists of patients submitted to imaging departments. The clinical management of hemophilia could be basically reinstructed on the basis of the information on early joint involvement provided by US by orienting appropriate prophylaxis regimen decisions on a personalized basis. The need for physiotherapy and/or specific recommendations about physical activity could be tailored on the objective evaluation of the joint status provided by this technique. Initial experience indicates a potential role of this technique in improving a patient’s understanding and awareness of joint abnormalities and in disclosing unexpected non-compliant patients.

## Figures and Tables

**Table 1:** Items included in different scanning protocols and scoring systems for ultrasound assessment of hemophilic arthropathy.

Author	Effusion (Synovial Fluid or Hemarthrosis)	Synovial Hypertrophy	Synovial Hyperaemia	Hemosiderin Deposition	Cartilage Abnormalities (Cartilage Loss, Hyperechogenicity, Thinning)	Bone Abnormalities (Erosion, Subcondral Cysts, Osteophytes)	Evaluated Joints
Klukowska 2001	YES	YES	YES	NO	YES	YES	Knee
Zukotynski 2007	YES	YES	NO	YES	YES	YES	Knee, ankle
Melchiorre 2011	YES	YES	NO	YES	YES	YES	Elbow, knee, ankle
Muça-Perja 2012	NO	YES	YES	NO	YES	YES	Knee, ankle
Martinoli 2013	YES ^1^	YES	NO	NO	YES	YES	Elbow, knee, ankle
Kidder 2015	NO	YES	YES	NO	YES	YES	Elbow, knee, ankle, hip, shoulder

^1^ The presence of intra-articular effusion is included in the scanning protocol but not considered in the scoring system because of its fluctuating nature. Adapted from [46]. Di Minno, M.N.; Ambrosino, P.; Quintavalle, G.; Coppola, A.; Tagliaferri, A.; Martinoli, C.; Rivolta, G.F. Assessment of hemophilic arthropathy by ultrasound: Where Do We Stand? *Semin Thromb Hemost*. **2016**, *42*, 541–549.
